# Black Truffle Aqueous Extract Attenuates Oxidative Stress and Inflammation in STZ-Induced Hyperglycemic Rats via Nrf2 and NF-κB Pathways

**DOI:** 10.3389/fphar.2018.01257

**Published:** 2018-11-09

**Authors:** Tongze Zhang, Muthukumaran Jayachandran, Kumar Ganesan, Baojun Xu

**Affiliations:** ^1^Food Science and Technology Program, Beijing Normal University-Hong Kong Baptist University United International College, Zhuhai, China; ^2^School of Biological Science, The University of Hong Kong, Pok Fu Lam, Hong Kong

**Keywords:** hyperglycemia, streptozotocin, oxidative stress, *Tuber melanosporum*, Nrf2, HPLC

## Abstract

**Background and Purpose:** *Tuber melanosporum* (black truffle) has been considered as a medicinal mushroom for a long time. *T. melanosporum* has the ability to attenuate oxidative stress and in turn diabetes mellitus (DM). DM has become an awfully common chronic unwellness, threatening people’s well-being. There are nearly 1 in 10 people in the world affected by diabetes. Oxidative stress plays a crucial role in vascular complications related to DM. Our study aimed to attain an effective treatment method to alleviate oxidative stress by scavenging free radicals and reducing inflammation, to display how truffle aqueous extract (TE) attenuates hyperglycemia.

**Methods:** Streptozotocin (STZ)-induced hyperglycemic rat model was accustomed to check the hypoglycemic effect of black truffle by relating it with Nrf2 and NF-κB pathways. Varied biomarkers and inflammatory markers were analyzed.

**Results:** Rats treated with TE showed reduced glucose levels, attenuated oxidative stress through regulation of SOD, CAT, VIT-E, and VIT-C. The gene expression of Nrf2 and NF-κB in rats treated with TE was increased to normal group level. The mRNA expression of inflammatory pathway genes and oxidative stress pathway genes in rats treated with TE was brought back normal. Similar results were achieved in the rats treated with standard drug, glibenclamide (GB). TE conjointly inhibits the state of inflammation within the tissues generally littered with the symptoms of hyperglycemia.

**Conclusion:** The results of our study show the hypoglycemic impact of black truffle on STZ-induced hyperglycemia in rats via Nrf2 and NF-κB pathways, and both pathways have significant improvement that may support the hypoglycemic impact of truffle.

## Introduction

Increasing prevalence and incidence rate have evidenced that diabetes mellitus (DM) remains a crucial threat to human health. It is estimated that 300 million people all over the world will be suffering from this metabolic syndrome by the end of 2025 (Smyth and Heron, 2006; Nicholson and Hall, 2011). Insulin secretion and action plays a crucial role in the onset of DM, it affects numerous metabolic pathways involving carbohydrates, fats, and proteins. Inadequate insulin secretion ends up in a condition referred to as hyperglycemia; further it provokes the oxidative stress and generates reactive oxygen species (ROS). ROS has the ability to destroy the integrity of different cells and it was often seen as the secondary complication of DM (Kaneto et al., 2010; Tahrani et al., 2011). Superoxide dismutase (SOD) is an antioxidant enzyme converting the superoxide radical into less potent H_2_O_2_ (Fukai and Ushio-Fukai, 2011). H_2_O_2_ was furthered scavenged with the assistance of another antioxidant enzyme catalase (Yamamoto et al., 1988). Apart from the enzymic antioxidants, vitamin C and E conjointly provides a biologically plausible mechanism to inhibit the free radicals. These antioxidant genes are typically regulated by the nuclear factor-E2-related factor 2 (Nrf2), which highly responds to the oxidative stress and migrate to the nucleus, and then further synthesize different types of endogenous antioxidants (Cullinan et al., 2003; Yamamoto et al., 2008).

Edible mushrooms are reported to have many bioactive compounds including phenols, nucleotides, polysaccharides, steroids, and terpenoids. All the compounds mentioned are proved to have various health benefits to human (Valverde et al., 2015). Mushrooms have been proved to have antioxidant abilities in some *in vitro* studies (Ribeiro et al., 2006; Islam et al., 2016). Nucleosides, nucleobases, and nucleotides existing in edible mushrooms can help in the regulation of many physiological processes via the pyrimidine and/or purinergic receptors (Jacobson et al., 2004). Nucleosides and nucleotides play vital roles in the metabolism of fatty acids, immune modulation, healthy gastrointestinal tract maintenance and even help to improve brain functions (Yamamoto et al., 1997; Schlimme et al., 2000). Since mushrooms are noteworthy sources of nucleosides, there is more attention paid to nucleoside contents in edible mushrooms (Chiriví et al., 2017).

A closely connected pathophysiological condition with oxidative stress is inflammation and is ascertained in DM (Dobrian et al., 2001). The pro-inflammatory genes together with cytokines, chemokines, and adhesion molecules are regulated through the NF-κB (Ho et al., 2000). Hence to seek out an active component which might treat the symptoms of DM, we chose *T. melanosporoum*, which is also known as black truffle. Black truffle has been used as an edible mushroom and a functional food by Europeans for a long time owing to its health-promoting impact. Some *in vitro* experiments showed that black truffle is rich in some specific phenolic acids (Yan et al., 2017), and phenolic acids are proven to have strong anti-cancer, anti-oxidant, and anti-inflammatory properties (Hollman, 2001; Ozcan et al., 2014).

Taking into consideration the above facts, we designed our experiment to seek out the ability of truffle extract to maintain glucose homeostasis, attenuate oxidative stress via regulating Nrf2 and numerous antioxidants and conjointly suppress the inflammation that was evidenced by numerous assays. The results of our study might provide a new candidate to check in depth within the field of diabetes management.

## Materials and Methods

### Chemicals

Diabetes inducing agent streptozotocin (STZ) and a standard drug glibenclamide (GB) were purchased from Sigma-Aldrich (Shanghai, China). The antibodies including nuclear factor kappa B (NF–κB), and nuclear factor erythroid 2 [NF-E2]-related factor 2 (Nrf2) were purchased from Abcam, China. All the primers used were designed using PubMed and obtained from Sangon Biotech (Guangzhou, China) (Table 1). The other chemicals used in the experiment were all purchased from Yuanye Biotechnology Co., (Shanghai, China).

**Table 1:** List of primers.

Gene	Forward	Reverse
Nrf2	TCTCCTCGCTGGAAAAAGAA	AATGTGCTGGCTGTGCTTTA
Keap-1	TCCATTGAAGGCATCCACCC	CTGGCAGTGTGACAGGTTGA
HO-1	TTAAGCTGGTGATGGCCTCC	GTGGGGCATAGACTGGGTTC
SOD	AGGGCGTCATTCACTTCGAG	CCTCTCTTCATCCGCTGGAC
NF-κB	ATCAATGGCTACACGGGACC	AGTTCATGTGGATGAGGCCG
TNF-alpha	ATGGGCTCCCTCTCATCAGT	TCCCTCAGGGGTGTCCTTAG
IL-6	AGCGATGATGCACTGTCAGA	TAGCACACTAGGTTTGCCGA

### Plant Material

*Tuber melanosporum*, which is commonly known as black truffle, was purchased in dry slice form from Yunnan Province (China) and used for the preparation of extract.

### Preparation of the Truffle Water Extract

The dry slice samples were ground to powder in order to increase the efficiency of extraction. The sample powder was twice pretreated with 70% ethanol in order to remove small molecular compounds, which can dissolve in ethanol. After being extracted by ethanol, the mushroom sample was mixed with DI water in the ratio of 1:5. The mixture was boiled for 2 h and the extract was collected and filtered. Rotary evaporator (Heidolph, Germany) was used to concentrate water extract to get a dry solid form for animal study.

### Experimental Animals

All the rats used in the experiment were male and belonged to the species albino Wistar. The body weight of the rats was in the range of 180–220 g. In the experiment, rats were acclimatized to the condition of 25°C and 40% humidity. Rats were settled in polypropylene cages with the 12 h light-dark cycle. Rats were allowed free access to drinking water and feed purchased from Southern Medical University, Guangzhou, China. The nutrient content of the feed was 21.1% of protein, 5.1% of fat, 60.0% of carbohydrates, 3.9% of fiber, 7.9% of minerals and 2.0% of vitamins.

During the first week of acclimatization, the rats were fed with standard rat chow and water. After the adjusting period, rats were induced to hyperglycemia by streptozotocin (STZ). The 0.1 M STZ solution was prepared with citrate buffer. The STZ was induced by intraperitoneal injection at a dose of 40 mg/kg b.w. After 24 h of injection, Accu-Chek commercial kit (Roche diagnostics plasma Mannheim, Germany) was used to determine the blood sugar level of the rats. Rats with fasting glucose level higher than 250 mg/dL were considered hyperglycemic rats.

### Experimental Timeline

Rats were separated into 5 groups (*n* = 6), there were 18 hyperglycemic rats and 12 normal rats in total. The truffle extract (TE) solution was prepared with DI water and the intragastric tube was used to administrate TE solution. The administration period lasted for 45 days.

Group I: Normal rats (control)

Group II: truffle extract control (normal rats administrated with 600 mg/kg b.w)

Group III: hyperglycemic control (STZ alone)

Group IV: hyperglycemic rats + truffle extract (400 mg/kg b.w)

Group V: hyperglycemic rats + GB (600 μg/kg b.w)

After the administration period, all the rats were fasted overnight and then 24 mg/kg b.w. of ketamine hydrochloride was injected (intramuscular) into the rats for anesthetization. A mixture of potassium oxalate and sodium fluoride (3:1) was added to the blood samples. The various tissues were dissected immediately and washed with ice-cold saline. After the dissection, all the organs were stored at −80°C.

### Characterization of Chemical Components in the Truffle Sample

#### Determination of Total Carbohydrate, Reducing Sugar, and Water-Soluble Polysaccharide

Determination of total carbohydrate was conducted by following the description of DuBois et al. (1956) and Krishnaveni et al. (1984) with some modifications. The content of reducing sugar was determined according to the DNS method described by Miller (1959) with little modification. The water-soluble polysaccharide was determined as follows: 1 g of truffle water extract was added into a conical flask with 60 ml of water and heated in boiling water for 3 h. Then the mixture was evaporated till around 20 ml of the liquid was left. The same amount of ethanol was added to the precipitated water-soluble polysaccharide. The slurry was centrifuged at 8000 rpm for 7 min and the water-soluble polysaccharide was collected and then dried at 60°C for 10 min to remove ethanol. The amount of dry matter collected was weighed and recorded as the amount of water-soluble polysaccharide.

#### Determination of Phenolic Compounds in Truffle Extract by HPLC

The phenolic acid content of the hot water extract of truffle was measured following Luthria and Pastor-Corrales (2006) method. A 0.5 g of dry extract was extracted thrice by using 50 ml of methanol/water/acidic/butylated hydroxytoluene (85:15:0.5:0.2) and then the tubes were shaken on an orbital shaker at a speed of 300 rpm for 3 h at room temperature. Then the solutions were transferred to centrifugation tubes and centrifuged for 20 min at the speed of 6000 rpm. Then the rotary evaporator (Heidolph, Germany) was used to concentrate the solution at 45°C. 2 ml of 25% methanol was used to dissolve the dry residue. An HPLC grade 0.2 μm filter was used to clean up the aliquot of the methanol sample.

The measurement of phenolic acids by HPLC followed the method of Xu and Chang (2009). A Waters (Milford, MA, United States) associate’s chromatography system with a 720 system controller, a model 7125 sample injector, a model 418 LC UV detector at 280 nm, and a model 6000 A solvent delivery system was used. A 4.6 × 250 mm, 5 μm, Zorbax Stable bond analytical SB-C18 column (Agilent technologies, Rising Sun, MD, United States) was used for separation at 40°C, and the temperature of the column oven was maintained at this temperature (Islam et al., 2016). The mobile phase comprised of solvent A (0.1% TFA) and solvent B (methanol), a flow rate of 0.7 ml/min and an injection volume of 20 μl. The good separation was found gradient elution; all peaks were identified, phenolic acids were quantified with a relative retention time of external standards. The phenolic acid contents were expressed as μg/mg on a dry weight basis.

#### Determination of Nucleobases and Nucleosides in Truffle Extract by HPLC

The contents of nucleobases and nucleosides were measured by the methods described by Ranogajec et al. (2010) and Zhang et al. (2012) with slight modification. Briefly, 1 g of truffle extract sample was dissolved in 15 ml of DI water. After dissolving properly, the liquid was filtered by 0.45 μm filter into 1.5 ml HPLC vial bottle. The prepared sample was ready for further analysis by HPLC. The standard stock solution was prepared by dissolving 3 mg of nucleobase or nucleoside standard (cytosine, cytidine, uridine, adenine, and guanosine) to 30 ml DI water together. Then the standard stock solution was diluted to different concentrations, the final concentrations of the series standard solutions were 10, 20, 40, 60, 80, 100 μg/ml. All the standard solutions were filtered through 0.45 μm filter and then transferred into HPLC vials. The mobile phase contained solvent A and solvent B. Solvent A was prepared by using K_2_HPO_4_ to adjust the pH value of 50 mM of the KH_2_PO_4_ solution to 5.8, and solvent B was HPLC grade methanol. Both Solvent A and B needed filtration and sonication for 30 min before use. The HPLC system used for determination was a Waters (Alliance System, e2695, Waters, Milford, MA, United States) with a UV-DAD detector (e2998, Waters, Milford, MA, United States) and separated using a C18 column (YMC-Pack ODS-AM-303, 4.6 × 150 mm, 5 μm particle size). The flow rate was set at 0.4 ml/min, and the injection volume of the sample and standard solutions was 10 μl. The detection wavelength was set at 254 nm. After the HPLC measurement, the nucleobases and nucleosides contents were calculated as μg/g on a dry weight basis.

### Biochemical Parameters

#### Estimation of Blood Glucose, Hemoglobin (Hb) and Glycated Hb (HbA_1_C)

Blood glucose, Hemoglobin (Hb) and glycated Hb (HbA_1_C) were estimated using commercial kits.

#### Determination of Antioxidant Enzymes

The activity of superoxide dismutase (SOD) was determined by the method of Weydert and Cullen (2010). Following the description of Beers and Sizer (1952), the activity of catalase (CAT) was measured.

#### Determination of the Non-Enzymic Antioxidants

The content of ascorbic acid (Vitamin C) was estimated following the method of Omaye et al. (1979). The content of Vitamin E was measured by the method of Baker et al. (1951).

### Immunoblotting

RIPA lysis buffer was used to homogenize the pancreas. Then, the homogenate was placed for 30 min on ice and followed by centrifugation at 4°C and 20,000 *g* for 20 min and the supernatant was used as a sample. Samples containing 50 μg of total protein were loaded and then separated on 10% SDS polyacrylamide gel electrophoresis. The SDS– PAGE gel was then transferred onto a PVDF membrane (Millipore) followed by electrophoresis. The blotting membranes were incubated with the blocking buffer containing 5% non–fat dry milk powder or 5% BSA for 2 h to reduce non–specific binding sites and then incubated with Nrf2, and NF–kB–p65 monoclonal (rabbit monoclonal; 1:250) and β–actin (rabbit monoclonal; 1:1000) in blocking buffer overnight at 4°C at a constant shaking. After this, membranes were treated/incubated with their respective secondary antibodies (anti-rabbit IgG conjugated to horseradish peroxidase) for 2 h at room temperature. After 2 h incubation blot membranes were washed with TBST buffer for 30 min. Immunoreactive protein was visualized with the help of chemiluminescence protocol (GenScript ECL Kit, Piscataway, NJ, United States and Image Quant LAS 500). Using a gel image study program the densitometric analysis of the gel was carried out. The obtained results were compared with the results of the standard protein β- actin.

### RNA Isolation and Semi-Quantitative Analysis of mRNA Abundance

Total RNA was extracted from the liver of rats using RNeasy mini kit (50) (Catalog Number.74104, Texas, United States) following the manufacturer’s protocol. For the reverse transcriptase-polymerase chain reaction (RT-PCR) analysis, first-standard of cDNA was synthesized from total RNA using cDNA synthesis kit (Invitrogen, United States) according to the manufacturer’s protocol and was used for polymerase chain reaction (PCR) amplification to estimate the expression of Nrf2, Keap-1, HO-1, SOD, NF-κB, TNF-α, and IL-6 were electrophoresed on a 1.0% agarose gel, and the bands were visualized with ethidium bromide (EtBr). The intensities of bands were captured with an Image Quant LAS 500 and quantified using Image Quant analyzer software. GAPDH was used as a loading control.

### Statistical Analysis

All data were denoted as the mean ± standard deviation (SD) of experiment numbers (*n* = 6). The one-way analysis of variance (ANOVA) was used to evaluate statistical significance using SPSS Version 15 (SPSS, Cary, NC, United States) and Duncan’s multiple range test (DMRT) was used to find individual comparisons. Values *p* < 0.05 is considered statistically significant.

## Results

### Contents of Phenolic Compounds in *T. melanosporum* by HPLC

The total phenolic content of Truffle water extract was found to be 1.72 mg/g gallic acid equivalent, which is comparable with several reports of total phenolic contents in another study on truffle mushroom. According to Villares et al. (2012), the phenolic contents of *T. aestivum*, *T. indicium*, and *T. melanosporum* were 1.88 mg/g, 1.52 mg/g, and 1.20 mg/g, respectively. The total phenolic contents can be varied between different species and origins of the samples (Al-Laith, 2010; Villares et al., 2012). However, the phenolic content is a combination of different compounds like hydroxybenzoic acids and hydroxyl cinnamic acids. Since total phenolic content measures by Folin-Ciocalteu method cannot provide information of composition of the phenolic content, HPLC was used to quantify individual phenolic acids. The samples and authentic standards were measured at 280 nm of wavelength because most of the phenolic compounds have maxima of absorption at this wavelength. A standard curve was made by the different concentrations of authentic standard, and the quantification was expressed as μg/g weight of the extract.

**FIGURE 1:**
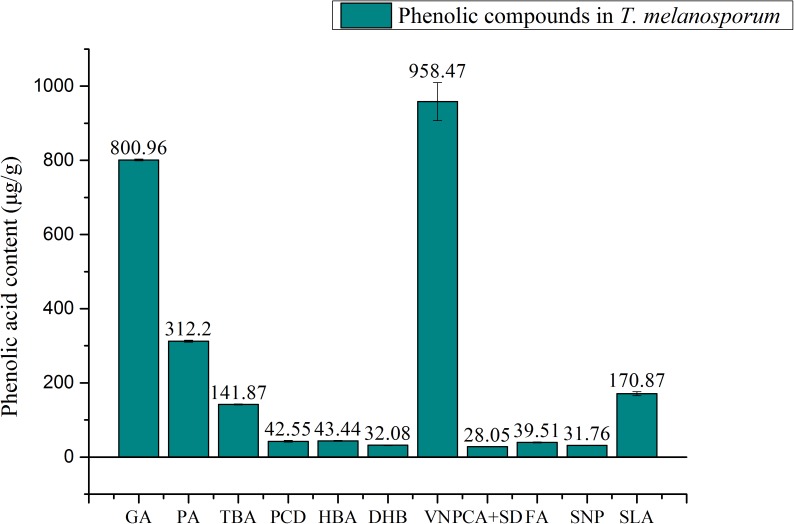
Quantification of phenolic compounds in *T. melanosporum* by HPLC; GA, gallic acid; PA, protocatechuic acid, TBA, 2,3,4-trihydroxybenzoic acid; PCD, Protocatechualdehyde; HBA, *p*-hydroxy benzoic acid; DHB, gentistic acid; VN, Vallin; PCA + SD, *p*-coumaric acid + syringaldehyde; FA, ferlic acid; SNP, sinapic acid; SLA, salicylic acid.

The quantification of phenolic compounds in *T. melanosporum* extract was presented in Figure 1. Fourteen phenolic compounds such as gallic acid, 3,4-dihydroxybenzoic acid, 2,3,4-trihydroxybenzoic acid, 3,4-dihydroxybenzaldehyde, 4-hydroxybenzoic acid, gentisic acid, chlorogenic acid, vanillic plus, caffeic acid, syringic acid, vanillin, *p*-coumaric acid plus syringaldehyde, ferulic acid, sinapic acid, and salicylic acid were analyzed. Among 11 detected phenolic acids, Vanillin (VN, 958.47 μg/g) were found to be the major in *T. melanosporum* extract followed by gallic acid (GA, 800.96 μg/g), protocatechuic acid (PA, 312.2 μg/g), salicylic acid (SLA, 170.87 μg/g), 2,3,4-trihydroxybenzoic acid (TBA, 141.87 μg/g), *p*-hydroxybenzoic acid (HBA, 43.44 μg/g), ferulic acid (FA, 39.51 μg/g), sinapic acid (SNP, 31.76 μg/g), gentisic acid (DHB, 32.08 μg/g), *p*-coumaric acid plus syringaldehyde (PCA + SD, 28.05 μg/g), and protocatechualdehyde (PCD, 42.55 μg/g). To compare with phenolic compounds in other mushrooms, gallic acid was reported for the first in *T. melanosporum* (Fernandez-Panchon et al., 2008; Kim et al., 2008; Palacios et al., 2011; Doǧan and Aydin, 2013; Hamza et al., 2016).

### Quantification of Nucleobases and Nucleosides in *T. melanosporum* by HPLC

**FIGURE 2:**
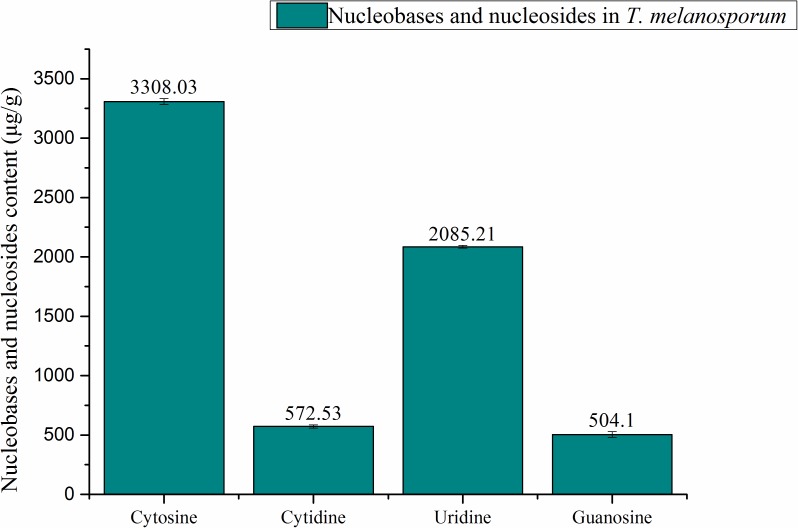
Quantification of nucleobases and nucleosides in *T. melanosporum* by HPLC.

A total of five standard compounds (adenine, cytosine, guanosine, cytidine, and uridine) were chosen to identify and quantify contents of nucleobases and nucleosides in TE. Samples and standards were measured at 254 nm, due to the maximum absorbance of nucleosides and nucleobases at that wavelength (Ranogajec et al., 2010). There was a total of 4 nucleobases and nucleosides found and quantified in TE sample. The quantification of nucleobases and nucleosides was shown in Figure 2. Cytosine (3308.03 μg/g) was found to have the highest content among all the nucleobases and nucleosides measured. The contents of uridine, cytidine, and guanosine were 2085.21, 572.53, 504.1 μg/g respectively. Compared with the previous study on nucleobases and nucleosides in fermentation mycelia of *T. melanosporum*, cytosine and cytidine were firstly found to have high contents in *T. melanosporum*. The uridine content in TE sample was higher than in the fermented mycelia sample of *T. melanosporum* (1671.9 μg/g). To compare with *T. indicum*, the Chinese local black truffle, the content of uridine was 7 times higher (Liu et al., 2011).

### Contents of Total Carbohydrate, Reducing Sugar, and Water-Soluble Polysaccharide

The contents of total carbohydrate, reducing sugar, and the water-soluble polysaccharide are shown in Table 2.

**Table 2:** Quantification of total carbohydrate, reducing sugar, and water-soluble polysaccharides.

		STD	Unit	Regression equation
Total carbohydrate	64.6	±2.58	mg/g	*y* = 0.0135x – 0.0073 (*R*^2^ = 0.9979)
Reducing Sugar	14.72	±0.34	mg/g	*y* = 0.0012x – 0.0602 (*R*^2^ = 0.9982)
Water-soluble polysaccharides	30	±1.2	mg/g	N/A

### Effect of TE on Blood Glucose

**FIGURE 3:**
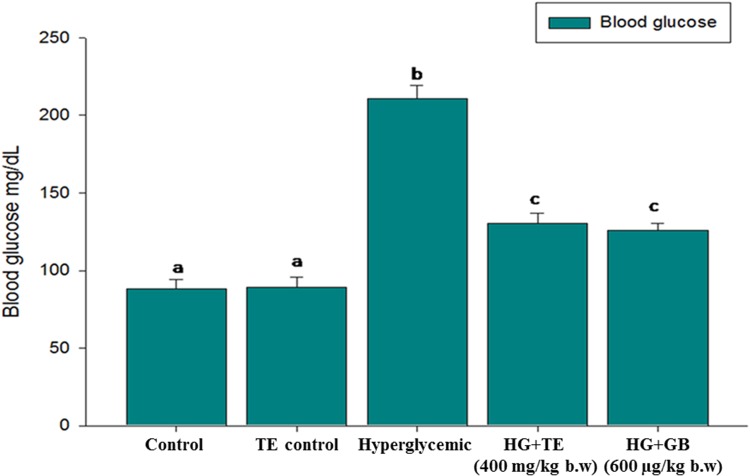
Effect of truffle extract on blood glucose. Each value is mean ± SD. of 6 rats in each group. In each bar, means with different superscript letter (a, b and c) differ significantly at *p* < 0.05 (DMRT). D: diabetic and GB- glibenclamide.

Hyperglycemia is an acute metabolic complication of diabetes mellitus, and it is one of the early symptoms of diabetes mellitus. Figure 3 elucidates the hyperglycemic condition of hyperglycemic rats, whereas on supplementation with TE 400 mg/kg b.w, the plasma glucose level was significantly (*p* < 0.05) reduced. Similar results were obtained in the GB treated rats. The results of TE and GB are not statistically significant with each other.

### Effect of TE on Hemoglobin (Hb) and Glycated Hb (HbA_1_C)

Several studies mention that low hemoglobin and high glycosylated hemoglobin levels were serious indicators of diabetes mellitus (diabetic nephropathy and retinopathy). Table 3 shows the effects of TE on hemoglobin and glycated hemoglobin, hyperglycemic rats showed an increased HbA_1_C and a decreased Hb, whereas in the treatment group the changes were significantly (*p* < 0.05) prevented compared to that of the hyperglycemic group (group 3). Similar kind of results was obtained in the standard drug-treated rats.

**Table 3:** Hemoglobin and glycated hemoglobin of the control and experimental rats.

Groups	Control	TE control	Hyperglycemic	HG + TE (400 mg/kg b.w)	HG + GB (600 μg/kg b.w)
Hemoglobin	15 ± 2^a^	15 ± 2^a^	10 ± 1^b^	12 ± 1^c^	12 ± 1^c^
Glycated hemoglobin	0.7 ± 0.2^a^	0.6 ± 0.2^a^	2 ± 0.3^b^	1 ±−0.3^c^	1 ± 0.3^c^

*TE, truffle extract; HG, hyperglycemic; and GB, glibenclamide. Values are given as means ± SD for six rats in each group. ^a^Indicates group (group 2) with no significance difference as compared to control group. ^b^Significantly different from control group at p < 0.05. ^c^Significantly different from the hyperglycemic group at p < 0.05. Duncan’s Multiple Range Test (DMRT).*

### Effect of TE on Enzymatic Antioxidants

Hyperglycemia initiates the production of free radicals beyond the capacity of the antioxidant system, which results in oxidative outburst. Table 4 shows that the concentration of antioxidative enzymes (SOD, and CAT) was decreased in hyperglycemic rats, whereas, on TE supplementation the levels of these enzymes were increased significantly (*p* < 0.05) as compared to the non–treated hyperglycemic group (group 3). Standard drug-treated rats showed similar changes equivalent to the treatment group, the results are statistically significant (*p* < 0.05) compared with the hyperglycemic group.

**Table 4:** Tissue enzymic antioxidant status of the control and experimental rats.

Groups	Control	TE Control	Hyperglycemic	HG + TE (400 mg/kg b.w)	HG + GB (600 μg/kg b.w)
SOD (50% NBT reduction/min/mg protein)					
Liver	8 ± l^a^	8 ± l^a^	4 ± 0.7^b^	7 ± 0.8^c^	7 ± 0.8^c^
Kidney	7 ± 0.8^a^	8 ± 0.8^a^	4 ± 0.6^b^	6 ± l^c^	6 ± 0.8^c^
Pancreas	6 ± 0.6^a^	6 ± 0.4^a^	3 ± 0.4^b^	5 ± 0.6^c^	5 ± 0.6^c^
CAT (μmoles of H_2_O_2_ utilized/min/mg protein)					
Liver	89 ± 6^a^	87 ± 6^a^	55 ± 4^b^	68 ± 5^c^	69 ± 6^c^
Kidney	44 ± 4^a^	45 ± 5^a^	24 ± 2^b^	32 ± 4^c^	32 ± 4^c^
Pancreas	26 ± 3^a^	25 ± 4^a^	9 ± 2^b^	14 ± 2^c^	15 ± 2^c^

*TE, truffle extract; HG, hyperglycemic; and GB, glibenclamide. Values are given as means ± SD for six rats in each group. ^a^Indicates group (group 2) with no significance difference as compared to control group. ^b^Significantly different from control group at p < 0.05. ^c^Significantly different from the hyperglycemic group at p < 0.05. Duncan’s Multiple Range Test (DMRT).*

### Effect of TE on Non–Enzymatic Antioxidants

Insulin sensitivity, protein glycosylation, and oxidative stress are the targets for the action of vitamin E and C. Table 5 shows that the concentration of Vit-E and Vit-C were decreased in the hyperglycemic rats, whereas, on TE supplementation these cellular antioxidants were increased significantly (*p* < 0.05) as compared to the hyperglycemic group (group 3). Standard drug-treated rats showed similar changes equivalent to the treatment group, the results are statistically significant (*p* < 0.05) compared with the hyperglycemic group.

**Table 5:** Tissue non-enzymic antioxidant status of the control and experimental rats.

Groups	Control	TE Control	Hyperglycemic	HG + TE (400 mg/kg b.w)	HG + GB (600 μg/kg b.w)
Vitamin C(mg/dL)					
Liver	2 ± 0.2^a^	2 ± 0.2^a^	1 ± 0.2^b^	1 ± 0.1^c^	1 ± 0.1^c^
Kidney	1 ± 0.1^a^	1 ± 0.1^a^	1 ± 0.1^b^	1 ± 0.2^c^	1 ± 0.1^c^
Plasma	2 ± 0.2^a^	2 ± 0.1^a^	1 ± 0.1^b^	1 ± 0.4^c^	1 ± 0.6^c^
Vitamin E(mg/dL)					
Liver	1 ± 0.1^a^	1 ± 0.1^a^	0.3 ± 0.1^b^	0.6 ± 0.1^c^	0.6 ± 0.2^c^
Kidney	1 ± 0.1^a^	1 ± 0.1^a^	0.2 ± 0.2^b^	1 ± 0.1^c^	1 ± 0.26^c^
Plasma	1 ± 0.09^a^	1 ± 0.2^a^	1 ± 0.1^b^	1 ± 0.1^c^	1 ± 0.2^c^

*TE, truffle extract; HG, hyperglycemic; and GB, glibenclamide. Values are given as means ± SD for six rats in each group. ^a^Indicates group (group 2) with no significance difference as compared to control group. ^b^Significantly different from control group at p < 0.05. ^c^Significantly different from the hyperglycemic group at p < 0.05. Duncan’s Multiple Range Test (DMRT).*

### Effect of TE on Protein Expression of Nrf2, and NF-κB

**FIGURE 4:**
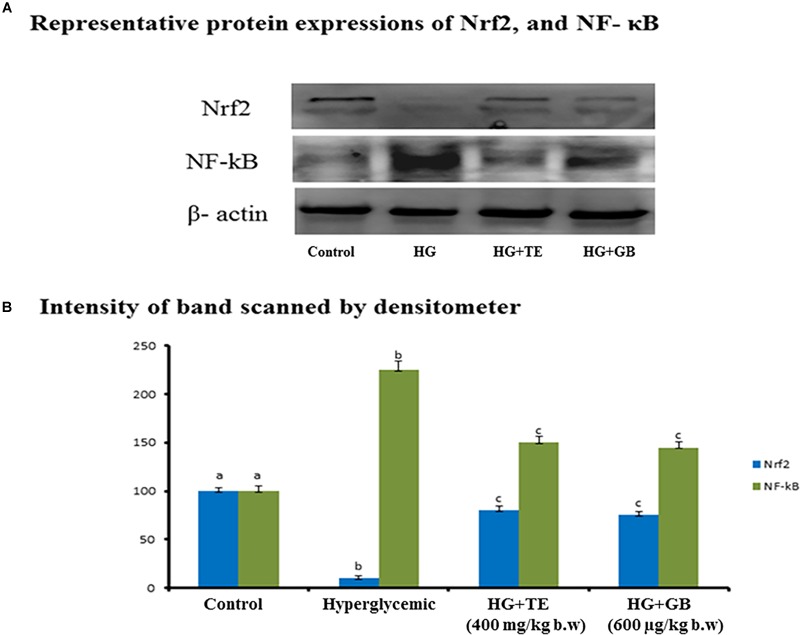
**(A)** Immunoblot quantifying nuclear factor (erythroid-derived 2)-like2 (Nrf2), and nuclear factor kappa B (NF-κB) expression. **(B)** Control group rats show normal protein expression and hyperglycemia rats (group 3) show increased expression of Nrf2 and NF-κB. Values are given as means ± SD for six rats in each group. ^a^Indicates group (group 2) with no significance difference as compared to control group. ^b^Significantly different from control group at *p* < 0.05. ^c^Significantly different from the hyperglycemic group at *p* < 0.05. Duncan’s Multiple Range Test (DMRT).

Nrf2 pathway regulates the expression of various antioxidant enzymes and several protein associates involved in this process, and which has a direct link with the insulin-signaling pathway. Since the outburst of oxidative stress results in severe inflammation and NF-κB is an important indicator of the inflammatory pathway. Figure 4 shows the effect of TE on Nrf2 and NF-κB protein expression. The expression of Nrf2 was significantly reduced in hyperglycemic rats when compared to control rats. Upon the supplementation with TE, these changes were significantly prevented and treated, and rats showed normal Nrf2 expression. Similar results were obtained in standard drug supplemented group rats. The expression of NF-κB was significantly increased in hyperglycemic rats as compared to the control rats and the expression was brought near to normal upon treatment with TE. Treatment with the standard drug showed changes equivalent to the treatment group, the results are statistically significant (*p* < 0.05) compared with the hyperglycemic group.

### Effect of TE on mRNA Expression of Inflammatory and Oxidative Stress Pathway Genes

**FIGURE 5:**
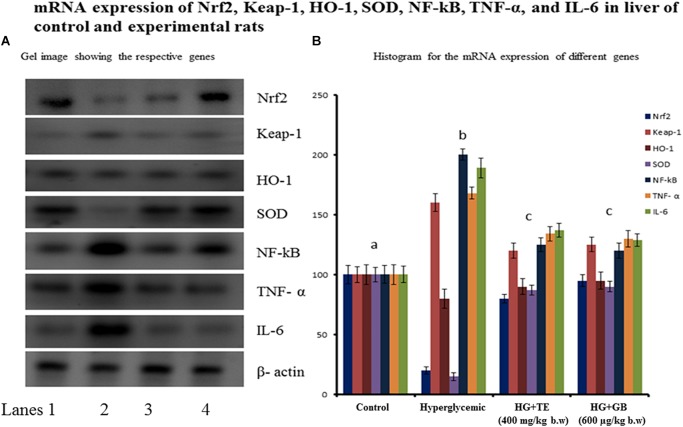
The mRNA expression of nuclear factor (erythroid-derived 2)-like2 (Nrf2), kelch like associated protein-1 (keap-1), hemeoxygenase-1, superoxide dismutase, nuclear factor kappa B (NF-κB), tumor necrosis factor- α, and interleukin-6 expression. Control group rats show normal protein expression and diabetic rats (group 3) show increased expression of Nrf2, keap-1, and NF-κB. **(A)** Lane 1- control, Lane 2- hyperglycemic rats, Lane 3- TE treated hyperglycemic rats, Lane 4- GB treated hyperglycemic rats. **(B)** Values are given as means ± SD for six rats in each group. ^a^Indicates group (group 2) with no significance difference as compared to control group. ^b^Significantly different from control group at *p* < 0.05. ^c^Significantly different from the hyperglycemic group at *p* < 0.05. Duncan’s Multiple Range Test (DMRT).

The gene expression regulates the protein translation of oxidative stress proteins and inflammatory pathway proteins are important to add strength to the existing biochemical parameters. Figure 5 shows the mRNA expression of Nrf2, Keap-1, HO-1, SOD, NF-κB, TNF-α, and IL-6. The expression of HO-1 and SOD were significantly reduced in hyperglycemic rats, and the TE treatment provided a chance to bring back the expression near to normal. The expression of Nrf2, Keap-1, NF-κB, TNF- α, and IL-6 were increased significantly in hyperglycemic rats and upon the treatment with TE restored its expression near to normal. Treatment with the standard drug showed similar changes equivalent to the treatment group, the results are statistically significant (*p* < 0.05) compared with the hyperglycemic group.

## Discussion

When the antioxidant competency of the cell is overwhelmed by the augmented production of reactive oxygen species as a result of oxidative stress it affects wide-ranging tissues. The oxidative stress is often seen related to inflammation in many dysfunctions, especially, DM (Muriach et al., 2014). In our study, hyperglycemia was induced using STZ (40 mg/kg b.w), STZ has the ability to preferentially affects pancreatic β-cells by causing toxicity. Once the STZ is taken up by the glucose transporter 2 of the pancreas, STZ induce diabetes via 3 different mechanisms: (1) methylation of DNA, (2) nitric oxide production, and (3) free radical generation. Hyperglycemia is a crucial indicator of DM, enriched glucose levels can also be a reason for the exaggerated free radical production (Matough et al., 2012). The combustion of glucose in the β-cells is tightly coupled to insulin secretion. The GLUT2 transporter plays an important role in the transport of glucose inside the β-cells, followed by glycolysis, Krebs cycle, and oxidative phosphorylation in order to synthesize ATP. The ATP/ADP ratio elevation, as a result of high blood glucose, leads to the closure of the KATP channel and opens the calcium channel on β-cell membranes. The calcium influx triggers insulin granules exocytosis and subsequent release of insulin. Upon administration of low dose of STZ, β-cells undergo severe necrosis and elimination by macrophages, the surviving or residual β-cells are unprotected to insistent hyperglycemia that can damage mitochondrial function in the lasting β-cell population. The low dose which we have used has the ability to bring a partial destruction of β-cells rather than the complete destruction of pancreatic β-cells (Yan and Wu, 2015).

These concepts are eventually observed from our experiments: the glucose levels of hyperglycemic rats were augmented considerably compared to control rats, upon treatment with TE the glucose level was kept in check and therefore the results are comparable to GB treated rats (Figure 3). We have chosen various tissues to investigate its antioxidants to determine the extent of oxidative stress. As a result of oxidative stress varied free radicals have been generated within the cell, however as the oxygen content is high in cells, therefore the formation of superoxide radical (O2 •-) becomes quicker (Dröge, 2002). Superoxide dismutase (SOD) performs the detoxification of superoxide radical into H_2_O_2_. SOD can be found in cells as different types based on the cofactor metal attached to it. The overall mechanism of action of SOD is referred to as a ping-pong mechanism; since it carries out consecutive reduction and oxidation of the metal center ultimately it produces H_2_O_2_ (Sheng et al., 2014). H_2_O_2_ is an additional harmful byproduct, which harms cells and tissues; therefore further detoxification method needed is carried by another vital inhibitor enzyme catalase. Cells often used catalase to readily catalyze the decomposition of hydrogen peroxide into water and molecular oxygen (Qujeq and Rezvani, 2007). Results of our study found that the levels of these enzymes were considerably reduced within hyperglycemic rats (group 3), as a result of augmented oxidative stress. Upon supplementation with TE the amount of those enzymes was augmented considerably as compared to the hyperglycemic rats. The messenger RNA expression of SOD conjointly provides results in favor of the biochemical studies (Figure 5), hyperglycemic rats show reduced mRNA expression of SOD and that was prevented within the treatment group (group 4) this could be due to the ability of TE to scavenge the free radicals and by that regulates the Nrf2 pathway. GB is typically the most used hypoglycemic drug and is taken into account as a standard drug for several experimental studies to compare the results achieved. Our results are compared with the standard drug-treated rats, the result of TE is almost similar to that of standard drug treated group and no significant difference was detected.

Vitamin C is a natural antioxidant and it has the structural similarity with that of glucose, which makes it a right candidate for diabetes. Vitamin C acts as a reducing agent in free radical-mediated oxidation process (Lobo et al., 2010); so, it will act as an antioxidant. Vitamin -E is a fat-soluble vitamin found in several foods, fats, and oils. It is conjointly an antioxidant that may help forestall harm to the body’s cells. Vitamin E supplements and other antioxidants could facilitate the scale back of the chance of heart disease and alternative complications in people with diabetes. Our study results show that the amount of these antioxidants was considerably reduced within the hyperglycemic rats (group 3), upon supplementation with TE, the amount of these antioxidants was improved considerably compared to hyperglycemic rats. The results obtained show there is no significant difference between the TE-treated and standard drug-treated rats.

The nuclear factor erythroid 2-related factor (Nrf2) is a transcription factor that functions as the key controller of the redox homeostatic gene regulatory network (Itoh et al., 1999). Kelch-like ECH-associated protein 1 (Keap1), a cysteine-rich protein that is anchored to actin within the cytoplasm, interacts with Nrf2, acting as an adapter protein for the Cul3-dependent E3 (Cul3) ubiquitin ligase complex. Beneath unstressed conditions, Nrf2 found hooked up to the complex and upon the influence of oxidative stress it tends to release from the complex and enters within the nucleus (Shelton and Jaiswal, 2013). In nucleus the free Nrf2 binds with antioxidant response element (ARE) in target gene sequence and transcribe the various antioxidants (NADPH) quinone oxidoreductase 1(NQO1), heme oxygenase 1 (HMOX1), glutamate-cysteine ligase (GCL) and glutathione S transferases (GSTs). The western blotting results showed varied regulation of these proteins within the hyperglycemic group (group 3). These changes were considerably prevented within the treatment group (group 4). The mRNA expression of HO-1 and different Nrf2 regulative genes are varied considerably in the hyperglycemic group, that was significantly prevented by the TE treatment and similar kind of results was detected in GB treated rats.

The nuclear transcription factor-κB was first found in β-cells and so subsequently found in all forms of cells (Lawrence, 2009). It plays key roles in cellular response to the outside and within stimulus, like free radicals, cytokines, stress, and viral and bacterial antigens. The NF-κB represents the Rel protein family. The NF-κB proteins contain a set of subunits, like c-Rel, RelA (P65), and RelB. NF-κB1 (P50) and NF-κB2 (P52) are synthesized from their larger precursors P105 and P100 (Oeckinghaus and Ghosh, 2009). NF-κB1 and NF-κB2 play key roles in the transactivation of target genes; they perform their functions by combining heterodimers with P65, RelB, and c-Rel. It plays a vital role in assessing the relationship between oxidative stress and inflammation. Results from our studies state that the expression of NF-κB and pro-inflammatory cytokines like TNF-α and IL-6 were markedly exaggerated within the hyperglycemic rats (group 3). Treatment with TE markedly reduced the expression of this protein (group 4). The reason could also be owing to the ability of TE to scavenge the radical and in association with the attenuation of oxidative stress, it conjointly inhibits the inflammation. The results of the TE-treated rats showed statistically similar results to that of GB treated rats and this could be considered for carrying out the experiment further. The results open up the field for the conversion of these experimental results into clinical trials. Commonly the doses vary species to species and the standard formula used to calculate the dose is (animal km factor divided by human km factor) multiplied by animal dose (mg/kg); the km factor, weight (kg) divided by BSA (m2).

## Conclusion

Overall, our results have given a concept to additionally focus on the hypoglycemic potential of truffle extract (TE) by evaluating varied alternative pathways. Results from this study have evaluated the effectiveness of TE to attenuate oxidative stress and the way it regulates the antioxidant synthesis at the molecular level. Our study conjointly finds the interrelation between the oxidative stresses, inflammation and focus it as the important pathogenesis condition in DM. Future studies on TE could offer a novel drug for the treatment of DM upon successful clinical trial.

## Ethics Statement

All the experiments were conducted in accordance with the principles provided by the National Institute of Health (NIH) guideline for the Care and Use of Laboratory Animals. The approval to carry out with this experiments was issued by the Animal Ethics Committee of Zhuhai Campus of Zunyi Medical University, China which also conforms to the Guidelines for Ethical Conduct in the Care and Use of Animals.

## Author Contributions

BX and MJ designed the study, carried out the experiments, and wrote, edited, and proofread the manuscript. TZ conducted the chemical characterization of mushrooms, the feeding and dissection of animals, and carried out the experiments and wrote the paper. KG helped with the feeding and dissection of animals.

## Conflict of Interest Statement

The authors declare that the research was conducted in the absence of any commercial or financial relationships that could be construed as a potential conflict of interest.
